# Supercritical CO_2_ Processing of White Grape Must as a Strategy to Reduce the Addition of SO_2_

**DOI:** 10.3390/foods12163085

**Published:** 2023-08-17

**Authors:** Cristina Cejudo, Ana Belén Díaz, Lourdes Casas, Enrique Martínez de la Ossa, Casimiro Mantell

**Affiliations:** Chemical Engineering and Food Technology Department, Wine and Agrifood Research Institute (IVAGRO), University of Cadiz, Puerto Real, 11519 Cadiz, Spain; cristina.cejudo@gm.uca.es (C.C.); lourdes.casas@uca.es (L.C.); enrique.martinezdelaossa@uca.es (E.M.d.l.O.); casimiro.mantell@uca.es (C.M.)

**Keywords:** SO_2_ alternative, preservation, PPO inactivation, antimicrobial effect, emerging technologies, high-pressure techniques

## Abstract

In winemaking, sulfur dioxide addition is the most common procedure to prevent enzymatic and microbial alterations. However, the enological industry looks for safer alternatives to preserve enological products, and high-pressure treatments with supercritical CO_2_ are a suitable alternative. This study evaluates the effectiveness of this process in the stabilization and preservation of white grape must, studying the influence of time, pressure, and CO_2_ percentage on must characteristics. In spite of the percentage of CO_2_ turned out to be the variable that affects the most the process, no remarkable differences were observed in pH, acidity, and color intensity between untreated and treated musts. Moreover, this technique has proven to be very efficient in the reduction of aerobic mesophilic microorganisms as well as in the reduction of residual polyphenol oxidase activities, being lower than those obtained with SO_2_ addition (60 and 160 mg/L). Based on the results, the most convenient conditions were 100 bar and 10% CO_2_, for 10 min treatment.

## 1. Introduction

In the productive process of white must or wines, it is necessary to prevent oxidative reactions, which cause the browning of the products and alter their chemical composition and sensory quality [1,2].

Sulfur dioxide (SO_2_) has been broadly used in the wine and juice industry, given its antimicrobial, antioxidant, and antioxidasic effects [3]. Actually, it is the most common treatment to prevent or reduce the proliferation of spoilage microorganisms. It is added to grape must before alcoholic fermentation, repressing non-Saccharomyces yeasts and promoting the rapid development of sulfite-tolerant Saccharomyces cerevisiae. It is also applied after fermentation and when bottling, as an antioxidant and preservative, helping to prevent oxidation reactions, which cause the browning of white musts and wines and change their chemical composition and sensory quality [1] as well as microbial contaminations [4]. In fact, SO_2_ acts by limiting the endogenous grape enzymes’ activity (polyphenol oxidase, tyrosinase, and peroxidase) and those that come from fungal infections (laccase), as well as reducing the effects of dissolved oxygen [5]. Despite its advantages, SO_2_ possess a risk to the health of sensitive individuals, such as headaches, allergies, and diarrhea and even its bioaccumulation could eventually lead to lung cancer [6]. In technological terms, it may be responsible for the development of sensory defects as well as for neutralizing the aroma of wines and juices [3]. For this reason, over the last two decades, there has been a growing trend in the winemaking industry to reduce the employment of sulfur dioxide and to seek alternative methods and practices that allow a greater reduction, or even its exclusion of obtaining products that satisfy consumer’s demands without compromising product quality along storage [7,8].

The International Organization of Vine and Wine (OIV) allows the use of other additives in winemaking such as dimethyl dicarbonate (DMDC) [9] or lysozymes to control microbial spoilage, or ascorbic acid to control oxidation. Although chemical additives generally provide some benefits from an economic point of view, additive techniques can alter the matrix and sensory properties of the product. Moreover, it is sometimes difficult to determine the optimal dose to be employed for these additives. Ascorbic acid, for instance, can act as a pro-oxidant if overdosed, so it should be used in combination with SO_2_ in order to remove the hydrogen peroxide that is formed during its oxidation [10]. Moreover, consumers show a steady trend to demand more natural products that include in their composition the lowest possible addition of preservatives. In this regard, non-thermal physical treatments may have the key to safe and quality food production and therefore have gained considerable importance recently [11,12]. High-ultrasounds [13], pulsed electric fields [1,14], or high-pressure technologies such as high-pressure homogenization [15], high-hydrostatic pressure [16,17,18], and cold plasma [19], are some of them.

High-pressure technologies have been listed by the US Food and Drug Administration as a suitable method to replace thermal pasteurization, as they are effective for the inactivation of enzymes in fruit-derived products [20], but there are only a few references using this technology in the oenological field. Ultra-high-pressure homogenization (3000 bar, 0.02 s) was applied to white grape must, leading to the total elimination of any initial microorganisms (1 × 10^6^ CFU/mL of wild yeasts and fungi and 7 × 10^3^ CFU/mL of bacteria) [21], leading to fruitier and more aromatic wines. Van Wyk et al. (2018) employed different preservation techniques for the production of red wine from the Cabernet Sauvignon variety, i.e., SO_2_, high-pressure processing, and pulsed electric fields [22]. Among these treatments, high-pressure processing (4000 bar for 5 s) was the only treatment that successfully prevented the growth of *Brettanomyces*, the most common cause of wine spoilage, avoiding the formation of off-flavors and off-odors over storage. Among the different high-pressure techniques, high-pressure CO_2_ (HPCD) is a great alternative, given that provides an inert environment that conveniently delays oxidation reactions. Moreover, it is able to inactivate a wide range of microorganisms and decrease the enzymatic load by employing mild temperatures and pressure levels above the supercritical region (Pc = 72.8 bar, Tc = 31 °C) [11]. This method has been demonstrated to be rather effective for the preservation of pomegranate, tomato, or strawberry juices among others with minimal changes in the sensory characteristic of aroma, color, and flavor of them respecting the fresh ones [23,24]. Marszałek et al. reported an increase in the shelf life of strawberry juice up to 20 days, by decreasing its enzymatic activity by 85% and preserving all of its chemical characteristics when treated at 45 °C and 300 bar for 30 min [24]. In addition, Oulé et al. reported maintenance of 88% of vitamin C after treatment of orange juice at 250 bar and 40 °C, preserving also their nutritional value [25]. With respect to oenologic processes, Izquierdo-Cañas et al. applied saturated CO_2_ to must as a strategy to reduce SO_2_ content in the prefermentative stage, taking advantage of the inert environment created by CO_2_, but those experiments were not carried out in a supercritical regime [26]. Those authors reported a good sensory evolution after 12 months of bottling, where CO_2_-treated wines in the absence of SO_2_ proved to be the most aromatic ones.

To the best of our knowledge, scarce papers have reported the supercritical CO_2_ treatment for the sterilization of grape must, and even less for its further winemaking. Therefore, this research intends to evaluate the effectiveness of a high-pressure process using supercritical CO_2_ for the stabilization of white grape must and present the treatment as an alternative to the use of SO_2_ to avoid oxidation and microbiologic contamination in winemaking. The variables time, pressure, and CO_2_ percentage will be optimized in order to evaluate the effect and interaction between them respecting the pH, total acidity, color, total polyphenols, antioxidant activity, and microbial inactivation.

## 2. Materials and Methods

### 2.1. Must Preparation

White grapes of Dominga variety were purchased from a local market and used as raw material. The must was obtained by pressing the grapes by means of a stainless steel 5 L laboratory scale vertical press. After that, the must was placed in 5 L methacrylate tanks and the commercial pectolytic preparation of Enovin Clar (Agrovin, Spain) was added to favor decantation (1.5 g hL^−1^). The debug was carried out at 4.5 °C for 24 h in the absence of light by covering the tanks with aluminum foil to avoid oxidation. The clear must was stored at −20 °C until use. The must obtained had a °Bé of 8.54 ± 0.11.

### 2.2. Supercritical Fluid Treatment

**Figure 1:**
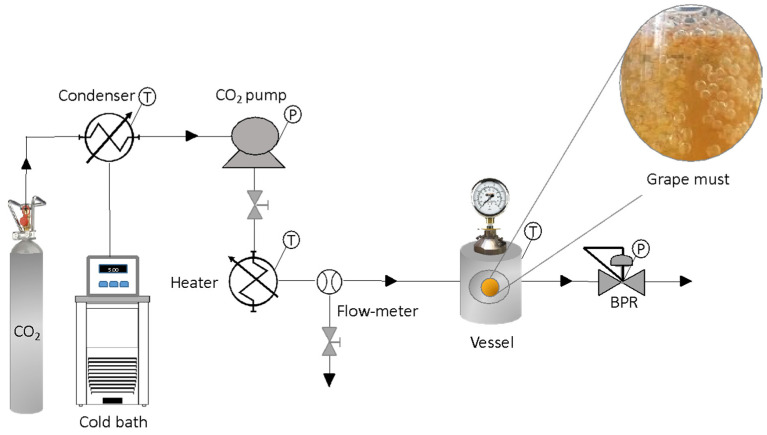
Flowchart of the supercritical fluid treatment.

The method described by Cejudo et al. [27] was followed with some modifications. The experiments were carried out in a high-pressure system supplied by Thar Technologies (Pittsburgh, PA, USA, model SF100), which consists of a 100 mL vessel fitted with a thermostatic jacket, a high-pressure pump, and a back-pressure regulator (BPR), to control the set pressure. The processing temperature was controlled by means of three thermocouples located on the vessel (inner and outer) and on the heater. The must was poured into the vessel and was hermetically sealed. The pressure was gradually increased at 10 g/min until the working conditions were reached. The system worked in batch mode, and the BPR remained closed over the whole treatment. When the processing time was completed, the BPR was slowly opened in steps of 5 bar, ensuring that the pressure gradually decreased inside the vessel until it reached atmospheric conditions. The must was recovered in aseptic conditions to avoid contamination, using sterile material and a Bunsen burner during must manipulation. The treated must were stored in sterile Falcon tubes at −20 °C until use. A descriptive flowchart of the process can be seen in Figure 1.

A multifactorial experimental design was carried out by executing a total of 12 experiments in duplicate, where the influencing parameters in the process have been evaluated (Table 1). The CO_2_ percentage (10, 40, and 70% (*v*/*v* of the vessel)), the pressure levels (100 and 250 bar), and the operating time (10 and 20 min) were the variables of the study. The temperature was maintained constant at 35 °C in order to work in a non-thermal-technique regime, avoiding any thermal conditions that might affect the organoleptic characteristics, while operating above the critical temperature of CO_2_.

After each treatment run, the vessel was washed off with water to remove any must residues and then disinfected by injecting ethanol through the system for 15 min.

### 2.3. Must Characterization

The parameters potentially susceptible to modification after the treatment were used as a variable response. During the treatment, CO_2_ is dissolved in water to form carbonic acid (H_2_CO_3_), which dissociates to bicarbonate (HCO_3_^−^), carbonate (CO_3_^2−^), and hydrogen ions [28]. Consequently, the pH and acidity of the must could be affected and had to be measured before and after the treatment. Moreover, the must color is an important factor with regard to consumers’ sensory and quality preferences. Color intensity has been used previously as an indicator to predict quality degradation when thermal treatment has been applied to fruits and vegetables [29]. Moreover, the measurement of color intensity is also related to the oxidation of white wines, indicating its quality [30]. For this reason, in this research, color intensity (absorbance at 420) was evaluated in grape musts before and after the supercritical CO_2_ treatment. Additionally, some parameters related to the biochemical properties of the musts, such as the antioxidant capacity and the total polyphenolic content have also been analyzed. Finally, in order to determine the suitability of the treatment for must stabilization, polyphenol oxidase activity and microbial inactivation have been determined.

#### 2.3.1. Oenological Parameters of the Musts

The pH of the musts was measured at 20 °C in duplicate by means of a digital pH meter (GLP 21; Crison; Alella, Spain). Total acidity analysis was analyzed following the method of Adolfo Lutz Institute (IAL 235/IV) [31]. A 40 mL sample (must:water 1:1) was analyzed by titration with standardized NaOH 0.1 M and adding phenolphthalein as an indicator. Each titration was carried out in duplicate, and the results were expressed as g/L of tartaric acid.

For color determination, the samples were filtered through a 0.45 μm nylon filter and the absorbance was measured at 420 nm using a UV–Vis spectrophotometer (Cary 60, Agilent Technologies, Santa Clara, CA, USA). Each measurement was carried out in duplicate.

CIELAB parameters (L*, a*, and b*) were measured using The Simplified Color Method for Wines of the free software MSCV^®^_7 [32], by measuring the absorbance at 450, 520, 670, and 620 nm. Along with the CIELAB parameters, this software provides an image of the approximated color of the samples based on these measurements.

#### 2.3.2. Total Polyphenol Index (TPI)

The analysis was conducted following the method described by Ribéreau-Gayon [33]. The samples were filtered through a 0.45 μm nylon filter, measuring the absorbance in a 1 cm path length quartz cuvette at 280 nm, using a UV–Vis spectrophotometer (Cary 60, Agilent Technologies, Santa Clara, CA, USA). The samples were previously diluted 1:10 with distilled water. Each determination was carried out in duplicate following Equation (1).

TPI = A280 × 10(1)

#### 2.3.3. Antioxidant Capacity

The antioxidant capacity of the musts was determined by means of a DPPH assay following the method described by Scherer and Godoy [34].

For the analysis, 0.1 mL of must was added to a test tube containing 3.9 mL of DPPH in an ethanol solution (6·10^−5^ M). After 24 h incubation at room temperature in the absence of light, the absorbance was measured at 515 nm and converted into the percentage of inhibition according to Equation (2):

(2) $$\%I=\frac{A_{0}-A_{i}}{A_{0}}\cdot 100$$

where A_0_ and A_i_ are the initial and the final absorbance of the sample, respectively.

#### 2.3.4. Polyphenol Oxidase Activity (PPO)

PPO activity was measured following the method described by Loira et al. [21] with some modifications. For the analysis, 1.5 mL of a solution of catechol (0.15 M) and 1.5 mL of sodium phosphate buffer (0.05 M, pH 6.5) were mixed with 450 μL of sample and incubated 24 h at room temperature (ca. 25 °C). The absorbance was measured at 420 nm.

One unit of enzyme activity (U) was defined as the amount of enzyme that causes a change of 0.001 in the absorbance level per minute. The residual activity (RA) of the PPO after high-pressure process with supercritical CO_2_ was calculated according to Equation (3):

(3) $$\mathrm{RA}\;\left(\%\right)=\frac{U_{\mathrm{Treat}}}{U_{0}}\cdot 100$$

U_Treat_ represents the enzyme activity registered after the treatment, while U_0_ represents the enzyme activity in the fresh grape must.

In order to assess the effectiveness of the supercritical CO_2_ treatment in PPO inactivation, results were compared to those obtained for the untreated musts and musts treated with SO_2_ at two concentrations (60 and 160 mg/hL of total SO_2_). All the analyses were carried out in triplicate.

#### 2.3.5. Microbiological Analysis

A microbial analysis was performed on treated and fresh must in order to evaluate the microbial inactivation after the CO_2_ treatment, by plate counting of the total aerobic mesophilic microorganisms and the total yeasts and molds [35,36]. For this purpose, the total aerobic mesophilic microorganisms were grown on plate count agar (PCA), composed of 4.5 g·L^−1^ tryptone, 2.25 g·L^−1^ yeast extract, 0.9 g·L^−1^ glucose and 13.5 g·L^−1^ agar. In addition, yeast and molds were grown on yeast extract peptone dextrose solid medium (YPD), composed of 10 g·L^−1^ yeast extract, 20 g·L^−1^ bacteriological peptone, 20 g·L^−1^ glucose and 20 g·L^−1^ agar and 100 mg·L^−1^ chloramphenicol. All agar plates were incubated at 30 °C for 48 h. Treated samples were pour-plated directly (100 μL), while serial decimal dilutions in a NaCl solution (9 g·L^−1^) were required for the fresh must.

The decimal reduction was calculated considering the initial and final microbial concentration after treatment. Moreover, in order to evaluate the efficacy of the CO_2_ treatment, the results were also expressed as a function of the decimal reduction time (D-value), which is the processing time required to reduce the initial microbial population by 90%. The microbial inactivation has been determined as the D-value (Equation (4)), where N_0_ is the CFU/mL of the fresh must, N is the CFU/mL of the treated must and t is the processing time.

(4) $${\mathrm{logN}}_{0}-\mathrm{logN}=\frac{1}{D}\times \Delta t$$

#### 2.3.6. Statistical Analysis

Both the experimental design and the analysis of the results were statistically analyzed using the STATGRAPHICS Plus 4.0 software. A univariate analysis of variance (LSD and ANOVA, *p* < 0.05) was selected to evaluate significant variables and factors.

## 3. Results and Discussion

### 3.1. Physico-Chemical Parameters

This section includes a comparative study of the physicochemical parameters (pH, acidity, and color intensity) of the untreated must against the treated must at different operating conditions. An analysis of the influence of these variables on the same physicochemical properties is carried out.

#### 3.1.1. Effects of the HPCD on the Treated Must

**Figure 2:**
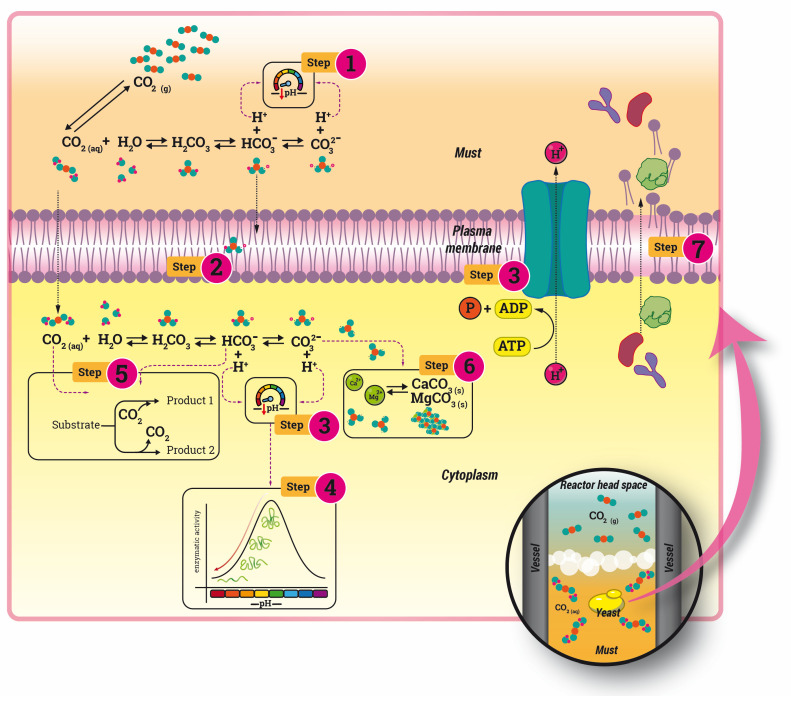
Graphical representation of the CO_2_ mechanisms of action over cells (based on [39]).

It has been previously reported the mechanisms of action of HCPD in complex matrices, describing the sterilizing effect as a succession of steps, which are represented in Figure 2 [37]:

1. The CO_2_ solubilization in the extracellular medium causes a decrease in the pH that contributes to the permeabilization of the membrane and the inhibition of microorganism growth.
2. The cell membrane modification, due to an easy penetration of CO_2_ through the lipidic membrane. At this point, CO_2_ can combine with the phospholipid layer creating cavities that cause a structural and functional disorder, increasing its permeability.
3. Penetration of the CO_2_ into the cytoplasm. The H^+^ protons generated are buffered in order to maintain the intracellular pH, but if the accumulation is too high, cells could be unable to maintain the pH homeostasis and the pH decreases.
4. Enzyme modification and metabolic interference for the pH decrease, causing cell death and decreasing enzyme solubility. Moreover, the reduction of the enzyme activity is linked to the denaturation phenomena due to the modification of their secondary and tertiary structure [38].
5. Metabolic interference of carbonic acid, involved in carboxylation and decarboxylation reactions.
6. Disorder of the electrolyte balance inside the cell. The carbonic acid inside the cell is converted to carbonate and causes Ca^+2^ and Mg^+2^ precipitation from cell membranes.
7. Extraction of constituents from the cell membrane and cytoplasm. CO_2_ increases the intracellular density and causes the dissolution of constituents, especially phospholipids and hydrophobic compounds, and transfers them to the extracellular environment.

**Figure 3:**
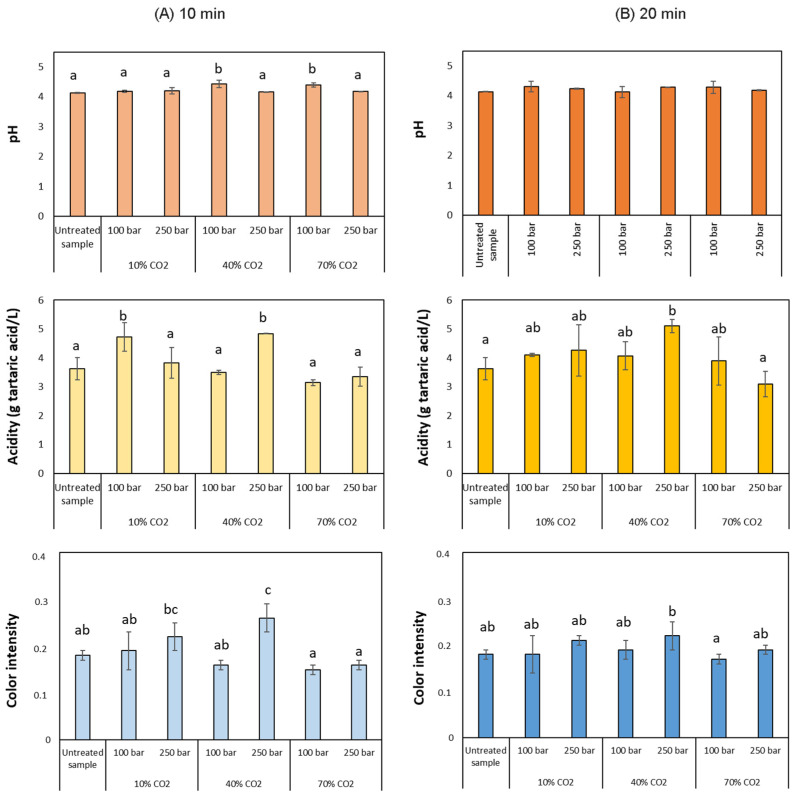
Physico-chemical characteristics of musts before and after the treatment. Different letters in each graph denote statistical differences (*p* < 0.05).

Although all those mechanisms have been observed, the complexity of the matrix causes the prevalence of one over the others. In this sense, the study of the characteristics of the must obtained could elucidate the sterilizing route. The chemical characteristics of musts before and after their supercritical CO_2_ treatment under different pressure levels, percentages of CO_2_, and processing time have been presented in Figure 3. The first bar in this figure represents the value reached by the untreated must, so that the effect of the treatment can be appreciated.

Any of the treated musts exhibited remarkably different values when compared to the untreated must. It could, therefore, be concluded that any of the configurations used would be appropriate for must treatment. According to the routes previously described, the pH modification seems to govern some of the changes. However, the treated samples’ pH did not seem to differ substantially from the control one, with the greatest variation being just 0.3 units. Considering the CO_2_ solubility in aqueous solutions and the density of CO_2_ at the conditions studied [40], the must introduced into the reactor is saturated in CO_2_ in all conditions, which could explain the low pH variations (Steps 1 and 3). Only two conditions, 100 bar at 40 and 70%, showed a pH statistically different from the control, showing any statistical difference among treatments when the operating time increased. Although the pH can suffer a substantial decrease given CO_2_ solubilization in aqueous solutions, it does not compulsory happen in complex matrices due to the buffer effect of other constituents [39]. Similar results were obtained by Silva et al. (2020) [13], who confirmed that CO_2_ did not significantly affect the pH values of apple juice submitted to very similar conditions to those used in the present study. Changes in pH and acidity have been reported after the application of supercritical CO_2_ as a result of CO_2_ dissolution into the liquid, forming carbonic acid that can be dissociated into bicarbonate and hydrogen ions (Step 1) [23,25]. However, in acid juices, the carbonic acid hardly dissociates into free hydrogen ions because the dissociation constant of carbonic acid and bicarbonate are pK_a_ = 6.57 and pK_a_ = 10.62, respectively [41]. Other authors also reported the transitory modification of the pH during the treatment, which could be attributable to the homeostatic function of the cells (Step 3) and the lower processing times.

On the other hand, depending on the operating conditions, acidity suffered slight variations. However, only significant differences were found on two conditions respecting the control at 10 min, which got reversed together with the pH at 20 min. In these two conditions, 100 bar and 10% CO_2_ and 250 bar 40%, acidity increases after treatment, which could contribute to the must grape preservation. It should be noted that although acidity can be adjusted during the prefermentative phase, this parameter is not only involved in the preservation of the must but also in the sensory perception of the final wine. In warm climate regions, acidity values can be an issue, since high temperatures during the harvesting season may result in lower acidity levels. A severe correction of acidity may contribute to a perception of a wine with edges and out of balance so this type of correction should be avoided to a great extent if a high-quality wine is to be obtained. On the contrary, higher %CO_2_ seemed to favor acidity reduction, although without significant differences at 70% CO_2_. Therefore, the variations seem to depend on the % of CO_2_ rather than on the pressure variable, since this latter parameter shows no clear influence on the response variable’s trends (Figures 3 and 5). The grape must used for these experiments had an initial acidity of 3.66 ± 0.39 g tartaric acid/L, being slightly lower than the typical acidity of other fruit juices, which may influence the result. It has also been reported the reduction of ascorbic acid concentration in orange juices treated with high-pressure carbon dioxide, although in that case without affecting significantly the total acidity compared to the control [42]. Izquierdo-Cañas et al. (2021) also observed a slight decrease in the acidity of grape musts saturated with CO_2_ when compared with the unsaturated control added with SO_2_ [26]. In no case, the acidity of the treated musts was lower than that of the control in the present work. This fact is interesting in winemaking, as acidity is a parameter that must be adjusted during the prefermentative phase to later carry out a correct fermentation process, particularly when wines are produced in warm regions.

Color represents one of the most important parameters for consumers’ acceptance [43]. Preference is given to minimally processed products with unaltered sensory properties because of detrimental manufacturing processes. As it is shown in Figure 3, there are some statistical differences regarding color intensity among samples, highlighting the 250 bar and 40% CO_2_ condition that differs the most from untreated control and the other operating conditions. Actually, the slight differences in color intensity that can be observed at shorter processing times, decreased after 20 min of processing time, showing very similar values to the control. Approximate color of untreated and treated must is included in Supplementary Materials.

These results agree with other published papers, where no significant changes have been reported regarding the color parameters of fruit juices (apple juice, orange juice, etc.), treated with HPCD technology [44,45]. For instance, chromaticity (C*) or color saturation was used to evaluate orange juice color changes after its treatment with supercritical CO_2_ (250 bar, 40 °C) compared to pasteurization (90 °C for 60 s) [25]. While untreated juice showed a value of 54.18, it slightly decreased to 52.84 after applying supercritical CO_2_, and it almost doubled (105.16) after pasteurization. This suggests that color changes are unlikely to be at the same level as when using thermal techniques.

**Figure 4:**
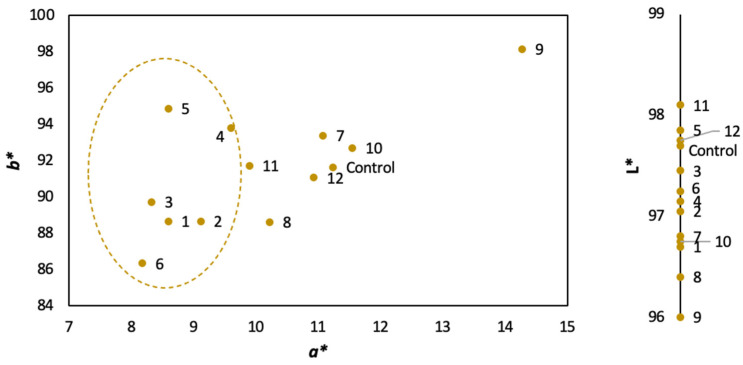
CIELAB coordinates of the control and treated samples.

Figure 4 shows the distribution of the samples according to the CIELAB coordinates. It can be seen how the pressure exerts its main influence on the a* coordinate contribution, as the musts treated at lower pressure values (trials 1–6 marked within the dotted line) are those with a more greenish perception, which agrees with the observations by the authors of apple juice studies [41]. Moreover, they also reported in the same study an increase in turbidity after the pressure processing of apple [46] and peach juices [47], which could be related to the slight decrease in luminosity that has been evidenced in this study. No clear trends were observed with regard to the b* coordinate.

#### 3.1.2. Determining the Influence of the Process Variables

**Figure 5:**
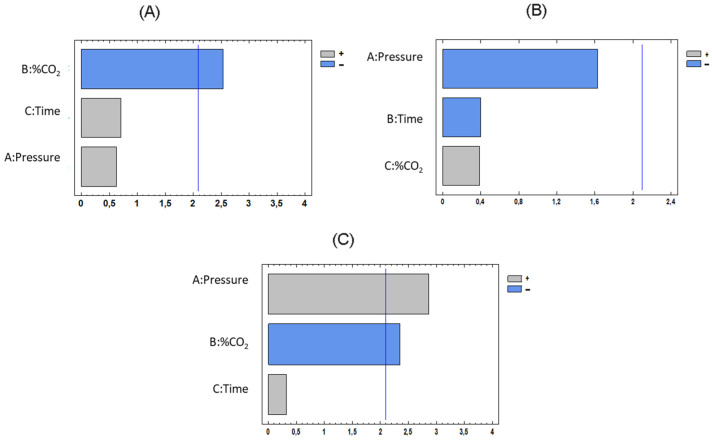
Pareto charts of the response variables: (**A**) acidity (**B**) pH, and (**C**) color intensity. The blue line indicates the limit from which the factors are considered statistically significant (*p* = 0.05).

In order to determine the influence of the operating factors (pressure, time, and %CO_2_), the multifactorial experimental design was evaluated by comparing the results of treated samples. Figure 5 shows the Pareto charts corresponding to each of the response variables at a *p* = 0.05.

By examining the experimental design, it can be confirmed that the time parameter was not a significant factor for any of the variables studied. In fact, no great variations of the physicochemical properties of the samples compared against those of the untreated must could be observed when different times were used (Figure 3). No effect attributable to this variable could be noticed even when the longest processing time in the study was used (20 min). This seems to be consistent with the previously reported results. Apparently, the changes in the matrix start to be noticeable after a certain processing time, even at higher processing temperatures than the one used in these experiments. For instance, when pomegranate juice was treated with supercritical CO_2_ at 45 °C and 127 bar for a longer time (40 min) [11], the pH remained unaltered. Furthermore, when apple juice was subjected to supercritical CO_2_ treatment at 65 °C, 100 bar for 40 min and 70 °C, 80 bar for 30 min, pH was not affected [48]. However, when apple juice was treated with supercritical CO_2_ at 45 °C, 200 bar for 60 min, not only did the pH of apple juice drop by 0.15 units but also noticeable differences in color after the treatment were observed [44]. As can be seen in Figure 5B and Figure S1, the conditions carried out in this experiment did not influence the pH of the musts.

As can be observed from Figure 3, total acidity varied when the operational conditions changed within the range studied, and was especially influenced by the %CO_2_ (Figure 5A). Regarding the absorbance at 420 nm of the treated and untreated musts, it increases at higher pressure values, which is not desirable in white grape musts (Figure 5C). In fact, when implementing other high-pressure processing techniques, pressure has been reported to be an influential parameter regarding the color changes in white grape must using other high-pressure processing techniques. Chang et al. (2017) reported higher color changes at higher pressure values when working in a range from 3000 to 6000 bar, where white grape juice was subjected to high hydrostatic pressure processing, although those color changes were of a lesser degree than those observed when employing conventional pasteurization [20].

According to the results observed, the %CO_2_ seems to be the variable that most affects the response variables since it exerts a significant influence on two of the three parameters studied, namely, acidity and color intensity. Even though the %CO_2_ is a significant process variable, the treated musts at 10, 40, and 70% CO_2_ did not show significant color differences with respect to the untreated must at *p* < 0.05 (Table 2). However, the musts treated at 70% CO_2_ showed a significant reduction of acidity with respect to the must treated at 10 and 40%, which makes these the least appropriate conditions for must treatment.

#### 3.1.3. Total Polyphenol Index and Antioxidant Capacity

**Figure 6:**
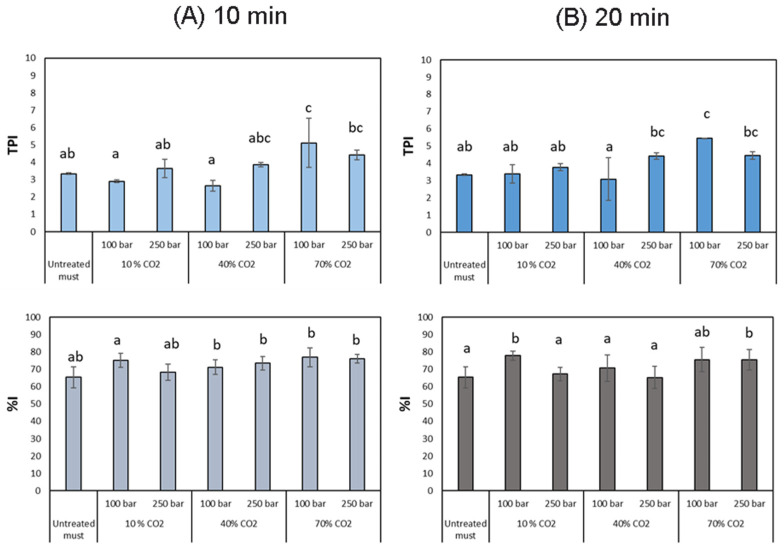
Total polyphenol index and antioxidant activity at (**A**) 10 min and (**B**) 20 min of treatment. Different letters in each graph denote statistical differences (*p* < 0.05).

Grapes represent an important source of polyphenolic antioxidants. However, their concentration, bioactivity, and bioavailability can be affected by many of the processing techniques that are used in food production [49,50]. When the proposed technology is employed on grape musts, none of the registered results from our experiments evidenced any drops in the phenolic compounds content or the antioxidant capacity of the musts. This indicates that both, phenolic compounds content and antioxidant properties, are preserved through the process, which agrees with the literature reports [51]. However, significant differences in total polyphenol index (TPI) started to be noticed as both CO_2_ percentages and pressure increased (Figure 6A). Other published works have reported an increment of TPI after extreme pressure treatments. For instance, an increase in the TPI by 15% was achieved when white grape juice was treated with high-pressure processing at 6000 bar for 3 min, while pasteurization would reduce this content by 4% [20]. Kaushik et al. (2014) obtained an increment of the total phenolic content in mango pulp by subjecting it to a high-pressure process at 6000 bar for 5 min [52]. In our particular case, the influence of the %CO_2_ on the TPI surpasses that of pressure, given that at low-pressure values this factor is not as influential as CO_2_.

Considering the saturation of the system in CO_2_, this behavior is probably explained by the capacity of CO_2_ to penetrate the cells and disrupt their membranes, forcing the release of their cytosol content (Steps 2 and 7). This effect seems to be enhanced by the higher density at 250 bar at 35 °C (901.95 g/L vs. 713.81 g/L at 100 bar), favoring both the solvating capacity and the diffusion into the cells. The slight increment in the phenolic compound content promotes the increment in antioxidant activity (Figure 6B), which is improved even to a higher extent than the TPI in time, due to the saturation of the musts with CO_2_ displacing the dissolved oxygen and eventually improving their oxidative stability.

### 3.2. Inertization Capacity of the Supercritical Treatment

Once it had been confirmed that high-pressure processing did not substantially alter the physical–chemical properties of the musts, the inertization capacity of the musts was determined.

#### 3.2.1. PPO Inactivation

Polyphenol oxidase belongs to a group of enzymes that catalyzes the oxidation of phenolic compounds that cause the browning of fruits and vegetables and alters their appearance and organoleptic properties [53]. It is, therefore, necessary to inhibit or at least reduce its activity in order to prevent the deterioration of the product over the processing and storage phases. In the present study, we have determined the efficiency of supercritical fluid treatments regarding the inactivation of the PPO by comparing its effect against the addition of SO_2_, since this is the most widely used treatment for preventing the oxidation of grape must.

**Figure 7:**
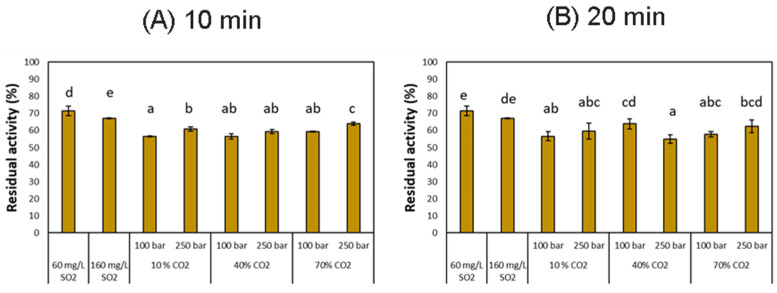
PPO inactivation of treated musts compared with SO_2_ addition at 10 min (**A**) and 20 min (**B**). Different letters in each graph denote statistical differences (*p* < 0.05).

Figure 7 shows the residual PPO activity in fresh and treated musts. In general, the residual activity of these enzymes was lower when employing supercritical treatment than when SO_2_ was added at two different doses, where 71.5 and 67.2% PPO residual activity could still be observed. This allowed us to confirm the high efficiency of the supercritical treatment. Differences were observed between the results obtained by the two doses of SO_2_ used and the treated musts, which lead us to think that the SO_2_ concentration to be added could be reduced in combination with the supercritical CO_2_ treatment. The maximum reduction of these enzymes’ activity was achieved at 100 bar, 10% CO_2_ and 10 min and 20 min, 100 bar, 40% and 10 min, and those at 250 bar, 40% CO_2_ and 20 min, with residual activities below 56.7%. These results let us think that possibly higher levels of pressure or higher percentages of CO_2_ than those considered in our study were needed to achieve a substantial inactivation of the enzymes.

The high efficiency exhibited by the treatment with supercritical fluids could be attributable to the action of the CO_2_ in an aqueous solution. Under these conditions, the CO_2_ causes the modification of the enzyme structure, affecting the tertiary and quaternary structures of the enzymes. At high-pressure values, water penetrates into the protein structure in a more efficient way, which seems to lead to protein unfolding, altering the enzymes’ stability [13] (Step 4). Furthermore, according to Li et al. (2014) [54], the inactivation of the PPO enzyme is related to the interaction between the CO_2_ and the hydrophobic active site of the enzyme, whose conformational and structural properties may change depending on the pressure and temperature conditions, leading to its inactivation. Here, the %CO_2_ influence is not critical in the results obtained, since not great differences are found in the ranges studied, probably due to the CO_2_ being saturated in the aqueous solution in all conditions [40], which is the form that intervenes in the modification of the enzyme structure. Therefore, a higher CO_2_ percentage does not lead to better inactivation, so it must be studied the CO_2_ proportion more suitable to achieve better results. For instance, Pozo-Insfran obtained a lower residual activity of PPO on grape must muscadine when using 7.5% CO_2_ than 15% [55].

On the other hand, the pressure seems to show a higher influence, and lower pressure values provided slightly lower residual activities, so the inactivation seemed to be enhanced at 100 bar. These results do not fit with those found in the literature. According to the bibliography, increasing pressure has a positive effect on enzyme inactivation. For instance, an activity reduction of 95% was achieved when apple juice was treated at 250 bar and 55 °C, which could be due to an increase in CO_2_ diffusivity at supercritical conditions [45]. For instance, Xu et al. (2011) reported the complete inactivation of the enzyme in apple juice at 220 bar, 60 °C for 10 min [56]. Moreover, when apple juice was treated at 100 bar, 45 °C for 30 min, only 32% of residual activity was measured [57] compared to that obtained at 600 bar, which achieved 20.8%. Nevertheless, the increase in temperature presumably offers better results in enzyme inactivation, so it seems that oxidases are relatively thermolabile enzymes. In this experiment, the temperature was maintained at a level close to the critical point, so it is considered a non-thermal technology, avoiding the possibility of alteration of organoleptic properties. At this temperature, the pressure parameter did not show great differences, so possibly working at higher pressure levels should provide more conclusive results about the sensitivity to the supercritical CO_2_ pressure.

#### 3.2.2. Microbial Inactivation

Supercritical CO_2_ technology has emerged as a promising non-thermal technology for the inactivation of pathogenic and spoilage microorganisms responsible for the spoilage of fruit and vegetable juices. In supercritical conditions, CO_2_ solubility improves its penetration into cells and membranes through physicochemical modification [11]. This fact produces the release of intracellular components, which are vital for the cell, causing microbial death [58] (Steps 2 and 7).

In this work, the initial microbial concentration of must was 9.5 × 10^3^ UFC/mL for yeast and molds, and 1.4*10^4^ UFC/mL for total aerobic microorganisms. The decimal reduction time (D-value) and cell reduction were used to evaluate the effectiveness of the microbial inactivation of the supercritical treatment with CO_2_ in the conditions tested (Table 3). D-value for the inactivation of molds and yeast could be just determined at milder conditions of 100 bar, 10% for 10 and 20 min, with values of 4.0 ± 0.8 and 6.7 ± 0 min, respectively, showing the influence on the processing time. From that condition on, any colony was observed in the YPD plates, so the growth of the microorganisms was under the limit of detection (<LoD). Thus, the pressure conditions have a high influence in the cell inactivation. These results would be in accordance with others reported in the bibliography. For instance, Ortuño et al. (2014) reported significantly higher decimal reduction reported by at 225 bar than at 100 bar at the time studied [38]. The starting point of microbial inactivation is influenced by the diffusion time of CO_2_ into the cells (Step 2), so the processing time has a great influence on this inactivation [13]. The diffusion capacity is influenced as well by the CO_2_ density, being at 35 °C and 100 bar 713.81 g/L and 901.95 g/L at 250 bar. It could be possible that the higher density of CO_2_ favors its penetration through the cell membrane, increasing the sterilization effect. In fact, the %CO_2_ contributes to the sterilization effect, showing comparable results at 70%CO_2_ at 100 bar than at any %CO_2_ but at 250 bar. This tendency has been also observed in other matrices, although the processing time also depends on other factors such as the pH of the juice. For instance, when hami melon (*Cucumis melo* L.) was treated with CO_2_ at 350 bar, the total microbial count was reduced when increasing the processing time from 5 to 60 min at 35, 45, and 55 °C [59]. A similar effect has been observed when pumpkin puree was treated with high-pressure CO_2_ at 275 bar between 1 and 8 h [60]. When pomegranate juice was treated with high-pressure CO_2_, the inactivation of mesophilic bacteria increased at higher temperatures and processing times at a fixed pressure (160 bar), reaching the maximum at 45 °C and 40 min.

The initial microbial load in this case remains at accepting levels, which eases the employment of low processing times. However, the initial contamination has to be considered in the establishment of the processing times [39].

In the winemaking process, the microbial inactivation of musts is of interest considering that *Brettanomyces dekkera* is one of the endogenous yeasts that can be found before the fermentation phase, causing the spoilage of the wine in the post-fermentation phases. Its inactivation would allow the implementation of the specific yeasts that promote the production of wines with particularly desirable organoleptic properties, which is especially important in natural wines with SO_2_ absence.

## 4. Conclusions

Supercritical CO_2_ as a non-thermal high-pressure technique has demonstrated its high efficiency to inactivate the microorganisms and enzymes in white grape musts while maintaining their bioactive and color properties. In general, no significant changes were observed in the studied response variables (pH, acidity, and color intensity) under the different evaluated ranges, when the treated musts were compared against the untreated must. Pressure and particularly the percentage of CO_2_ were the most influential factors on some of the response variables, while time did not exhibit any relevant influence on the resulting product. With regard to the chemical characteristics of the musts, the acidity was maintained or even statistically increased with respect to the control must at certain operating conditions, which could contribute to the must grape preservation.

In summary, the most appropriate conditions for the treatment of grape must with supercritical CO_2_ were 100 bar, 10% CO_2_, for 10 min, since low pressure and CO_2_ percentage values were not only more efficient but also more economically viable and less detrimental to the chemical characteristics of the treated musts in comparison with those of the fresh untreated must. Although the time parameter was shown as a non-influential factor in the modifications of the chemical properties of the musts, it seems to have an effect on the microbicidal effect, which is mandatory to consider at higher scales, since the diffusion and preservative effect of CO_2_ would require longer processing times.

On the other hand, the treatment exhibited a positive influence on the total polyphenol index, antioxidant capacity, and enzymatic inactivation of the musts, as well as a biocide activity against spoilage microorganisms. According to the results obtained, this process stands out as a remarkable alternative to the addition of SO_2_, or as a suitable method to reduce the amount of SO_2_ in grape must for winemaking production, as well as for its direct commercial use, producing more natural and safer enological products.

## Figures and Tables

**Table 1:** Conditions used for each experimental run.

Experiment Number	P (bar)	% CO_2_	V_sample_ (mL)	t (min)
1	100	10	90	10
2	100	10	90	20
3	100	40	60	10
4	100	40	60	20
5	100	70	30	10
6	100	70	30	20
7	250	10	90	10
8	250	10	90	20
9	250	40	60	10
10	250	40	60	20
11	250	70	30	10
12	250	70	30	20

**Table 2:** Multiple comparisons as a function of %CO_2_ according to Fisher’s least significant difference mean test.

	Color Intensity	Acidity
Untreated must—10% CO_2_	nd	nd
Untreated must—40% CO_2_	nd	nd
Untreated must—70% CO_2_	nd	nd
10% CO_2_–40% CO_2_	nd	nd
10% CO_2_–70% CO_2_	*	*
40% CO_2_–70% CO_2_	*	*

The asterisks (*) indicate statistically significant different contributions (*p* < 0.05); nd: no differences detected.

**Table 3:** Decimal reduction (log UFC/mL) of molds and yeasts after treatment.

Pressure	% CO_2_	Time	Log UFC/mL Reduction	D-Value (min)
100	10	10	2.6 ± 0.5	4.0 ± 0.8
	10	20	2.9 ± 0.0	6.7 ± 0
	40	10	3.5 ± 0.7	5.9 ± 1.2
	40	20	<LoD	<LoD
	70	10	<LoD	<LoD
	70	20	<LoD	<LoD
250	10	10	<LoD	<LoD
	10	20	<LoD	<LoD
	40	10	<LoD	<LoD
	40	20	<LoD	<LoD
	70	10	<LoD	<LoD
	70	20	<LoD	<LoD

## Data Availability

All the data presented in this study are available in this article.

## Supplementary Materials

The following supporting information can be downloaded at: https://www.mdpi.com/article/10.3390/foods12163085/s1, Figure S1: Approximate color of untreated and treated musts given by the free software MSCV^®^.
