# Supplementary material for: Supercritical CO2 Processing of White Grape Must as a Strategy to Reduce the Addition of SO2
Source: Foods. 2023 Aug 17;12(16):3085. doi: 10.3390/foods12163085 (PMC10453421; doi:10.3390/foods12163085)
Supplement: Supplementary file 1 [file foods-12-03085-s001.zip › foods-2531530-supplementary.pdf]

SUPPLEMENTARY MATERIAL

Trial	P (bar)	%CO_2	t (min)	Approximated color
Untreated must				
1	100	10	10	
2	100	10	20	
3	100	40	10	
4	100	40	20	
5	100	70	10	
6	100	70	20	
7	250	10	10	
8	250	10	20	
9	250	40	10	
10	250	40	20	
11	250	70	10	
12	250	70	20	

Figure S1. Approximate color of untreated and treated musts given by the free software MSCV®.
